# Titanium(III, IV)-Containing Catalytic Systems for Production of Ultrahigh Molecular Weight Polyethylene Nascent Reactor Powders, Suitable for Solventless Processing—Impact of Oxidation States of Transition Metal

**DOI:** 10.3390/polym10010002

**Published:** 2017-12-21

**Authors:** Vladislav A. Tuskaev, Svetlana C. Gagieva, Dmitrii A. Kurmaev, Nikolay A. Kolosov, Elena S. Mikhaylik, Evgenii K. Golubev, Alexander I. Sizov, Sergey V. Zubkevich, Viktor G. Vasil’ev, Galina G. Nikiforova, Mikhail I. Buzin, Olga A. Serenko, Boris M. Bulychev

**Affiliations:** 1 Department of Chemistry, M. V. Lomonosov Moscow State University, 1 Leninskie Gory, 119992 Moscow, Russia; sgagieva@yandex.ru (S.C.G.); dmitrykurmaev@mail.ru (D.A.K.); kolosovna@mail.ru (N.A.K.); aisizov@yandex.ru (A.I.S.); zubkevich.sergey@gmail.com (S.V.Z.); b.bulychev@highp.chem.msu.ru (B.M.B.); 2 A. N. Nesmeyanov Institute of Organoelement Compounds, Russian Academy of Sciences, 28 ul. Vavilova, 119991 Moscow, Russia; mihachenok@yandex.ru (E.S.M.); jeckagolubev@gmail.com (E.K.G.); Viktor@ineos.ac.ru (V.G.V.); ggn@ineos.ac.ru (G.G.N.); buzin@ineos.ac.ru (M.I.B.); oserenko@yandex.ru (O.A.S.); 3 Enikolopov Institute of Synthetic Polymer Materials, Russian Academy of Sciences, Profsoyuznaya Str., 70; 117393 Moscow, Russia

**Keywords:** Ziegler–Natta polymerization, coordination compounds, titanium, ultrahigh molecular weight polyethylene, mechanical properties

## Abstract

Catalytic systems containing TiCl_4_ or TiCl_3_, THF, organomagnesium (*n*-Bu_2_Mg) and organoaluminum compounds capable of producing ultrahigh molecular weight polyethylene (UHMWPE) were developed. The resulting polymers were characterized by a molecular weight in the range of (1.8–7.8) × 10^6^ Da and desirable morphology, suitable for modern methods of polymer processing—the solvent-free solid-state processing of superhigh-strength (tensile strength up to 2.1 GPa) and high-modulus (elastic modulus up to 125 GPa) oriented films and film tapes. The impacts of a THF additive, the oxidation state of the titanium atom, and the composition and nature of the nontransition organometallic compounds on the formation of catalytic systems for UHMWPE production were evaluated. The results indicate the suitability of individual titanium chloride tetrahydrofuran complex application for the formation of THF-containing catalytic systems. This approach also results in a significant increase in the system catalytic activity and mechanical properties of UHMWPE. The catalysts based on Ti(III) were inferior to systems containing Ti(IV) in productivity but were markedly superior in the mechanical properties of UHMWPE.

## 1. Introduction

Ultrahigh molecular weight polyethylene (UHMWPE) is a perspective engineering polymer that is widely used in demanding applications [1]. Because of its high melt viscosity, the polymer cannot be processed via conventional methods (i.e., injection molding, blow molding, extrusion, etc.). By reducing the entanglement density, the UHMWPE nascent reactor powders can be processed in solid-state below their melting temperature, leading to the high-strength and high-modulus oriented materials. This methodology, which was begun by Smith et al. [2,3], received considerable development by Rastogi et al. [4,5]. However, not only metallocenes [6] or nonmetallocene catalysts [4,5,7,8] allow us to obtain UHMWPE with a reduced number of entanglements.

Joo et al. reported that the catalytic system consisting of TiCl_4_, Bu_2_Mg, nonstoichiometric amounts of THF, and two types of organoaluminum compounds—Et_3_Al and Et_2_AlCl—allows the synthesis of UHMWPE with molecular weights in the range of (1.0–3.8) × 10^6^ under mild conditions (at 10–60 °C and ethylene pressures of 1 to 3 atm, solvent—petroleum ether). Polymer film tapes obtained using the solvent-free solid-phase monolithisation of reactor powders (below melting point) had a tensile strength of 1.7–2.0 GPa [9,10,11].

Despite the fact that Ziegler–Natta (ZN) catalysts, containing THF, are well studied and are widely used in industry, the true role of THF in the formation of the catalytic system remains the subject of discussion. For example, titanium- and vanadium-containing ZN catalysts, which include THF complexes of MgCl_2_, are highly active in ethylene polymerization [12,13] and its copolymerization with α-olefins [14,15] resulting in ultrahigh molecular weight polymers.

One of the possible functions of THF in titanium–magnesium ZN catalysts is the modification of MgCl_2_, formed by the reaction (1) as described by Kissin et al. [16]. 

(1) R_2_Mg + 2Et_2_AlCl = 2MgCl_2_ + 2Et_2_AlR

Reaction of MgCl_2_ with a Lewis base, typically alcohols or ethers such as THF, is a generally known method of its activation [17,18,19,20].

Alternatively, THF can serve as a ligand interacting with TiCl_4_. Such interaction would convert the purely Ziegler type catalytic system to a nonmetallocene type. This possibility is illustrated by Sautet et al. [21], who described the formation of Ti(IV) alkoxo-complexes due to the THF ring opening in the presence of a strong Lewis acid-Ti(IV) species. This information indicates that the multivariance of chemical processes that may occur in the system {TiCl_4_ + R_2_Mg + THF + Et_3_Al + Et_2_AlCl} will inevitably lead to a certain nonreproducibility of the results and difficulties in the control of the catalytic process.

**Figure 1:**
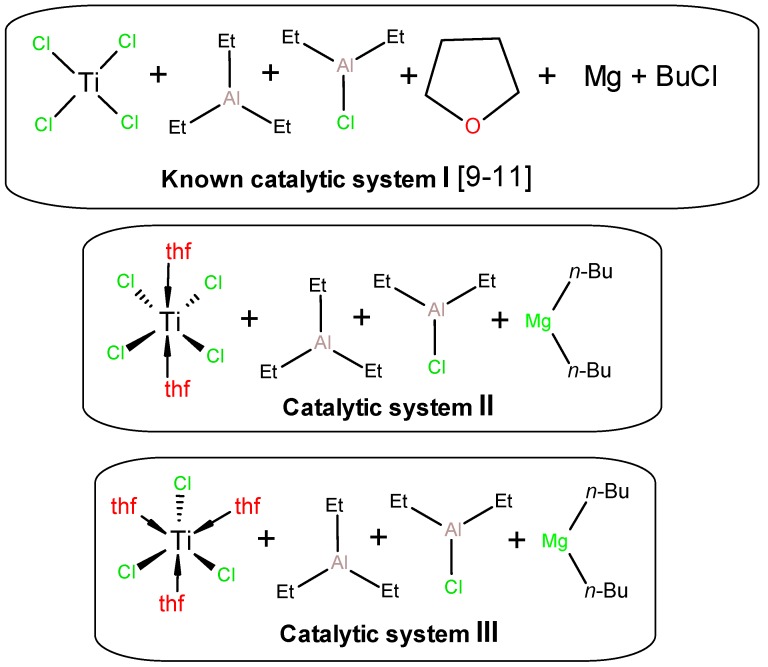
Compositions of catalytic systems **I**–**III** used in the present study. The exact ratio of activators for each experiment is given in Table 1.

Undoubtedly, the basic THF molecule will react with all inorganic and organometallic compounds of the system which are sufficiently strong Lewis acids. The key question is which of the formed products will have the greatest impact on the catalytic activity and properties of the polymer. It was previously demonstrated that in the presence of electron-donor organic ligands, such as THF, acetonitrile, or crown-ethers, ionic binuclear complexes of different composition can be formed. The precise composition of such complexes depends on the oxidation state of Group 4 metals and differences in acidity of electron-deficient molecules that are introduced to the system. Thus, the authors [22,23] have shown that titanium (or zirconium) in the formal +4 oxidation state in the presence of magnesium compounds is always included in the anion: [Mg(CH_3_CN)_2_(^15^C_5_)]^2+^[Ti^IV^Cl_6_]^2−^, [Mg(thf)_6_]^2+^[Zr^IV^CI_6_]^2−^, [Mg(thf)_6_]^2+^[Zr^IV^Cl_5_(thf)]^2−^ [Mg(thf)_6_]^2+^[TiCl_5_(thf)]^−2^, [(thf)_4_Mg(μ-CI)_2_Ti^IV^CI_4_] and [Mg_2_(μ-Cl)_3_(thf)_6_]^+^[TiCl_5_(thf)]^−^. At the same time, titanium (III) chloride in the presence of aluminum compounds is always included in the cation, e.g., {[(Ti^III^Cl_2_)(15-crown-5)]^+^[AlCl_4_]^−^} [24]. The above-mentioned behavior of titanium compounds in different oxidation states, in our view, is of fundamental importance to understanding the mechanism of system **I** (Figure 1) in terms of catalytically active site formation. Thus, it is expected that the formation of chlorine-containing titanium(IV) (Zr, Hf) anions will withdraw a transition metal atom from the catalytic process. In contrast, formation of a titanium-containing cation will promote the formation of catalytically active centers, corresponding to the most common point of view regarding their nature [25,26,27]. In other words, if the qualitative and quantitative composition of the cocatalyst will be constant, we can assume that the catalytic activity of the system in ethylene polymerization will be determined by the structure of the cationic active site containing Ti(III) with some optimal amount of THF.

The aim of this article is twofold. The first is to develop and optimize a cheap and technologically acceptable catalytic system capable of producing UHMWPE nascent reactor powders which will be processed via a solid-state solventless method. Secondly, we aim to examine the impact of a THF additive, the oxidation state of the titanium atom, and the composition and nature of nontransition organometallic compounds on the productivity of the catalytic systems, the properties of UHMWPE powders, and the specific features of plastic deformation during the orientational drawing of the compacted and monolithized material in the film tapes.

## 2. Materials and Methods

Toluene, hexane, and THF were distilled from Na/benzophenone prior to use. The water content of these solvents was periodically tested using Karl Fischer coulometry with a Methrom 756 KF apparatus. Argon and ethylene of special-purity grade were dried by purging through Super Clean™ Gas Filters (Zoetermeer, The Netherlands). Diethylaluminumchloride, aluminium sesquichloride, di-*n*-butylmagnesium, triethyl aluminium (Sigma-Aldrich, St. Louis, MO, USA) were used as 1.0 M solution in heptane. Tetrahydrofuran complexes of Group 4 transition metal chlorides TiCl_4_·2THF, TiCl_3_·3THF, ZrCl_4_·2THF, and HfCl_4_·2THF were synthesized according to known methods [28,29]. All manipulations with air-sensitive materials (precatalysts, organoaluminum compounds, Bu_2_Mg) were performed with rigorous exclusion of oxygen and moisture in oven-dried Schlenk glassware on a dual manifold Schlenk line, interfaced to a high-vacuum line. 

The polymerization of ethylene was carried out in a jacketed 100 or 300 mL stainless steel reactor (Parr Instrument Co., Moline, IL, USA) equipped with a mechanical stirrer, a temperature controller, and inlets for loading components of catalytic systems and ethylene. The reactor was kept under vacuum for 1 h at 50 °C before each experiment, after which it was filled with argon and cooled to the required temperature. An appropriate solvent (petroleum ether, b.p. 70–80 °C, or toluene) and the necessary amount of a cocatalyst and additives (for example, Et_3_Al (0.8 mL of 1 M solution in toluene, 8 × 10^−4^ mol), Bu_2_Mg (0.6 mL of 1 M solution in toluene, 6 × 10^−4^ mol), THF (0.008 mL, an aliquot in toluene), Et_2_AlCl (6 mL of 1 M solution in toluene, 6 × 10^−3^ mol) were loaded into the argon-purged reactor and stirred vigorously. Depending on the system, precatalysts were activated with Et_2_AlCl, Et_3_Al_2_Cl_3_, Et_3_Al, iBu_3_Al, methylaluminoxane (MAO), and Bu_2_Mg in various combinations; the composition of the activator mixtures for each experiment is indicated in Table 1. The reactor was heated to a specified temperature and the reaction mixture was saturated with ethylene to achieve a total ethylene and solvent vapor pressure of 0.7 atm. Polymerization was initiated by the addition of precatalyst to the reaction mixture. The pressure of ethylene was maintained constant during polymerization; the temperature was thermostatically controlled as indicated. After a desired period of time, the reactor was vented. Polymerization was stopped by the addition of 10% HCl solution in ethanol to the reactor. The polymer was filtered out, washed several times with a water–ethanol mixture, immersed in acidified ethanol for 12 h to remove inorganic impurities, and then dried under vacuum at 50–60 °C until a constant weight was achieved.

### Polymer Evaluation Methods

DSC was performed using a differential scanning calorimeter DSC-822e (Mettler-Toledo, Greifensee, Switzerland) at a heating rate of 10 °C/min in air. TGA and DTA measurements were done using a “Derivatograph-C” (MOM, Budapest, Hungary) at a heating rate 10 °C/min in air. The viscosity average molecular weight of the synthesized UHMWPE samples was calculated with the Mark–Houwink equation: *M_W_* = 5.37 × 10^4^ [η]^1.37^ [1], where *M_W_* is the viscosity average molecular weight (g/mol); [η] is the intrinsic viscosity in decalin at 135 °C (dL/g); [η] = (2η_sp_ − 2*ln*η_r_)^1/2^/0.056 (η_sp_ is the specific viscosity decalin at 135 °C; η_r_ is the relative viscosity in decalin at 135 °C; η_r_ = η_sp_ + 1).

The mechanical characteristics of the oriented materials were evaluated on the oriented tapes obtained by solid-state processing of the UHMWPE nascent reactor powders. Monolithic tapes (100 microns in thickness and 10 mm in width), uniform over the entire length, were formed at a pressure and shear deformation below the polymer melting point (124–126 °C). The tapes were subjected to uniaxial drawing using Spinline Daca equipment (Shanghai, China). The drawing temperature was set 4 °C below the polymer melting point. The mechanical characteristics of the tapes were measured with a Hounsfield H1KS machine (Redhill, UK) at the gauge length of the tested samples (120 mm) with a 2 mm/min initial deformation rate. The reported values are the average of at least eight samples.

Scanning Electron Microscopy investigations on morphologies of nascent reactor powders were carried out with a high resolution Tescan Vega 3 (Carl Zeiss Leo 1530 VP, LaB6, Oberkochen, Germany) operated at 15 kV. As-polymerized particles were carefully deposited on SEM stubs, and the samples were coated with gold using a sputtering technique.

## 3. Results and Discussion

When testing UHMWPE synthesis using the catalytic system {TiCl_4_ + R_2_Mg + THF + Et_3_Al + Et_2_AlCl} (**I**), proposed by Joo et al. [9,10,11], we used published data, but, for technical reasons, have introduced some modifications. As such, we used commercial di-*n*-butylmagnesium, reduced the amounts of reactants and the solvent volume proportionally to the reactor volume, and increased the TiCl_4_ concentration to 4 times the original concentration to overcome the constraints when adding the reagent. The components used for the catalytic system formation are presented in Figure 1.

**Figure 2:**
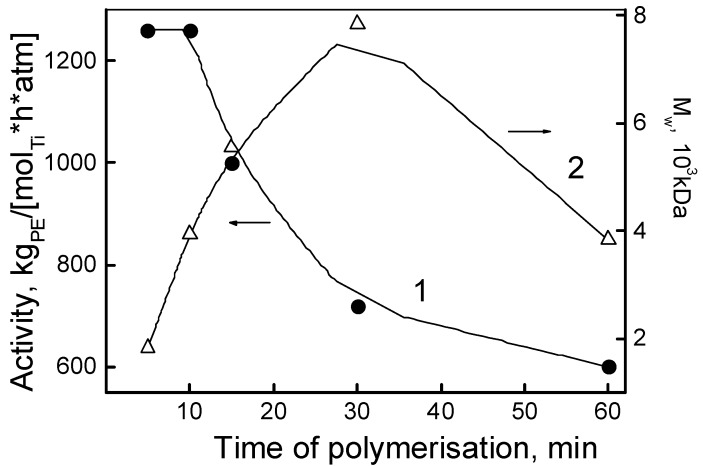
Catalytic activity (1) of the system **I** and molecular weight of the obtained ultrahigh molecular weight polyethylene (UHMWPE) samples (2) versus the polymerization time.

Figure 2 shows the kinetic dependences of the activity of the catalytic system **I** and the *M_W_* of the resulting PE. From the 15 min mark onwards, a monotonic decrease in activity was observed, and at 60 min it reduced twofold (Figure 2; Table 1, entries 1–5). Simultaneously, within the time interval of 5 to 30 min, the molecular weight of the polymer increased from 1.83 × 10^3^ to 7.84 × 10^3^ kDa (Figure 2, curve 2), and then decreased to 3.5 × 10^3^ kDa at 60 min. Therefore, during that same interval of time (5–30 min) the catalytically active sites are characterized by homogeneity providing a living or “pseudo-living” polymerization regime, but only for a given time interval. Based on this observation, all subsequent polymerization experiments were conducted for 30 min.

In order to elucidate the impact of the titanium oxidation state and the amounts of THF and nontransition organometallic compounds in the polymerization of ethylene, we used different catalytic systems, the composition of which is given in Table 1. Their properties were compared with the catalytic system **I** described by Joo et al. [9,10,11]. It is not possible to use other known catalysts for comparison with the considered catalytic systems, since commercial supported ZN catalysts do not give PE with the desired morphology (i.e., such polymers cannot be processed by a solventless method). On the other hand, single-site catalysts (metallocenes or phenoxyimine complexes of titanium) are MAO-activated catalysts but MAO has not shown sufficient efficiency on our systems.

**Figure 3:**
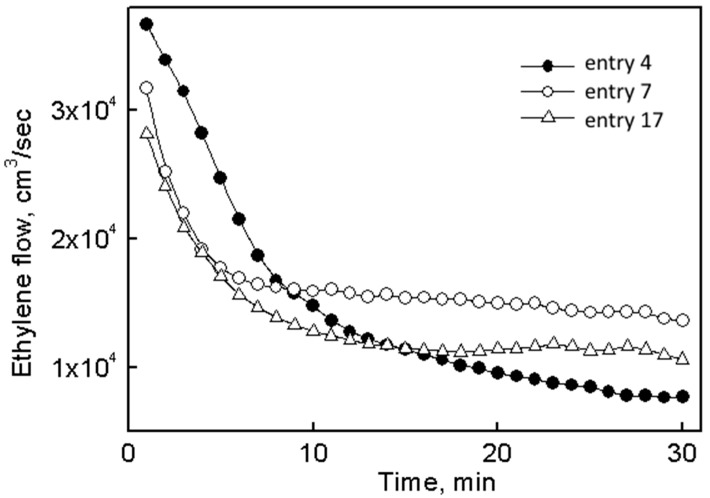
Kinetic dependence of catalytic systems based on catalytic systems **I** (entry 4), **II** (entry 7) and **III** (entry 17).

Application of system **II** allowed an approximately twofold reduction of the concentration of titanium compared with system **I** (entries 6–12). Moreover, application of activators in a ratio Et_2_AlCl/Et_3_Al/Bu_2_Mg (300:40:30) increased the activity of the catalytic system by ~2.5 times (entry 7 vs. entry 4); its resistance to deactivation was also slightly improved (Figure 3, curves 1 and 2). 

Generally, both activity and stability of nonmetallocene systems increase when the aliphatic solvent is replaced by toluene (e.g., [30,31]). The systems evaluated in this work display similar behavior (e.g., entries 6 and 7); this may be due to the limited solubility of tetrahydrofuran complexes of group 4 metal chlorides in petroleum ether. For this reason, all further experiments were carried out in toluene.

The introduction of an additional amount of THF into the system **II**, significantly exceeding complex stoichiometry, led to a sharp decrease in its activity. Thus, in entry 8, conducted under the same quantities of cocatalysts but with large amounts of THF, the system activity decreased almost threefold. This can be explained, for example, by the formation of inactive or much less active heterometallic magnesium complexes with anions [TiCl_6−*n*_·THF*_n_*]^−*x*^, similar to that previously described [23,24].

A twofold decrease in the concentration of Et_2_AlCl results in a considerable decrease in activity (entry 9 vs. 7), whereas the complete elimination of Et_3_Al from the catalytic system **II** has practically no influence on its activity (entry 10 vs. 7). TiCl_4_·2THF, activated by the mixture of Et_2_AlCl and Bu_2_Mg in a ratio of 150:50, showed good activity (1320 kg PE/mol Ti·h·atm, entry 11). It is interesting that the replacement of Et_2_AlCl for Et_3_Al_2_Cl_3_, which usually leads to increased activity of nonmetallocene systems (for example, [32]), in our case led to its decrease by one-third (entry 11 vs. 12, Table 1).

The activation of catalytic systems **I** (entries 21–22, Table 1) and **II** (entries 13–14) by methylaluminoxane (MAO) or triisobutylaluminium (TIBA) in the Ti/Al molar ratio 1:300 is accompanied by a sharp drop in the activity and molecular weight of the obtained polymers. The morphology of the reactor powders also undergoes negative changes, which, in particular, is manifested in a marked increase in the bulk density.

Attempts to form catalytic systems similar to **II** but using ZrCl_4_·2THF or HfCl_4_·2THF or equimolar mixtures of ZrCl_4_·2THF and TiCl_4_ THF were either unsuccessful or had no effect on the heterometallic system activity (entries 15–16). This is not surprising given the results of all early works on Ziegler catalysis, starting with metallocene and nonmetallocene systems containing groups 4 and 5 metals, as well as a significantly higher reduction potential M^4+^/M^3+^ for zirconium and hafnium compounds (for metallocene Cp_2_MCl_2_: −*E*_12_ = 1.4 (Ti), 1.6–2.3 (Zr) and 2.7 (Hf) [33,34,35] and titanium and zirconium nonmetallocene compounds with a pyrone ligand: −*E*_1\2_ = 0.48 (Ti); 1.3 (Zr)) [36].

Thus, the results discussed above clearly indicate the involvement of Ti(III) in the formation of catalytic systems initially containing TiCl_4_ or TiCl_4_·2THF. As can be seen from Table 1, the properties of the catalytic systems **III** formed from TiCl_3_·3THF, with composition shown in Figure 1, behave directly opposite to systems based on TiCl_4_ 2THF. For example, the presence of Et_3_Al in the system with TiCl_3_·3THF increases its activity (entry 17 vs. 19), and the use of aluminum sesquichloride instead of Et_2_AlCl does not lead to its fall (entry 18 vs. 20). 

**Figure 4:**
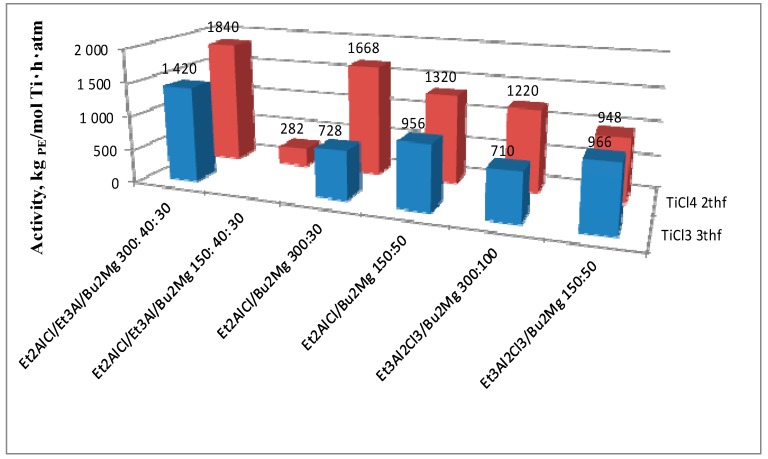
Activities of catalytic systems with TiCl_3_ and TiCl_4_ tetrahydrofuran complexes versus the composition of the activation mixture.

Catalytic system **III** was fairly active without the Et_3_Al cocatalyst (entries 18–20), but, in most cases, it was inferior to system **II** (Figure 4). In some cases, the ZN catalysts formed in situ (systems **I** and **II**, entries 1–16, in our opinion) showed superior results to systems obtained from single components [37,38]. As seen from Table 1, the presence of Et_3_Al in mixture (ceteris paribus) led to a significant increase in activity of the catalyst based on TiCl_3_·3THF (entry 17 vs. 19), which suggests that its role is not limited to the reduction of Ti(IV) to Ti(III), but is necessary for the effective alkylation of the transition metal. 

### Properties of Reactor Powders

All obtained polymers lack branching at the main chain, which is evident from the absence of an IR absorption band at 1378 cm^−1^, characteristic of the symmetric deformational vibrations of CH_3_ groups. The bulk density of the obtained UHMWPE reactor powders varies in the range from 0.05 to 0.09 g/cm^3^. The melting point and degree of crystallinity are typical for UHMWPE [1,39] and are in the range of 141–149 °C and 58–78% respectively (Table 1). Explicit differences in the characteristics of reactor powders of polymers obtained using catalytic systems of different compositions were not found.

**Figure 5:**
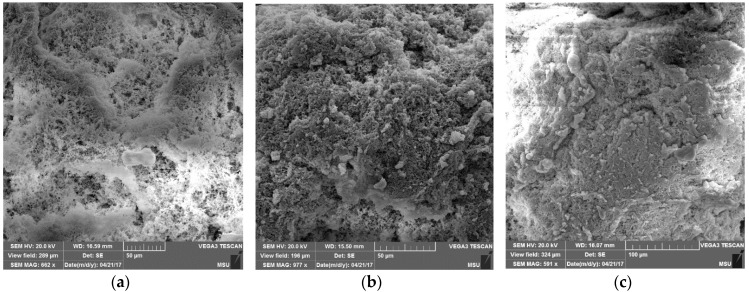
SEM images of the surface morphology of PE powders obtained from the catalytic system **I** for 10 min (**a**), 15 min (**b**), and 30 min (**c**).

**Figure 6:**
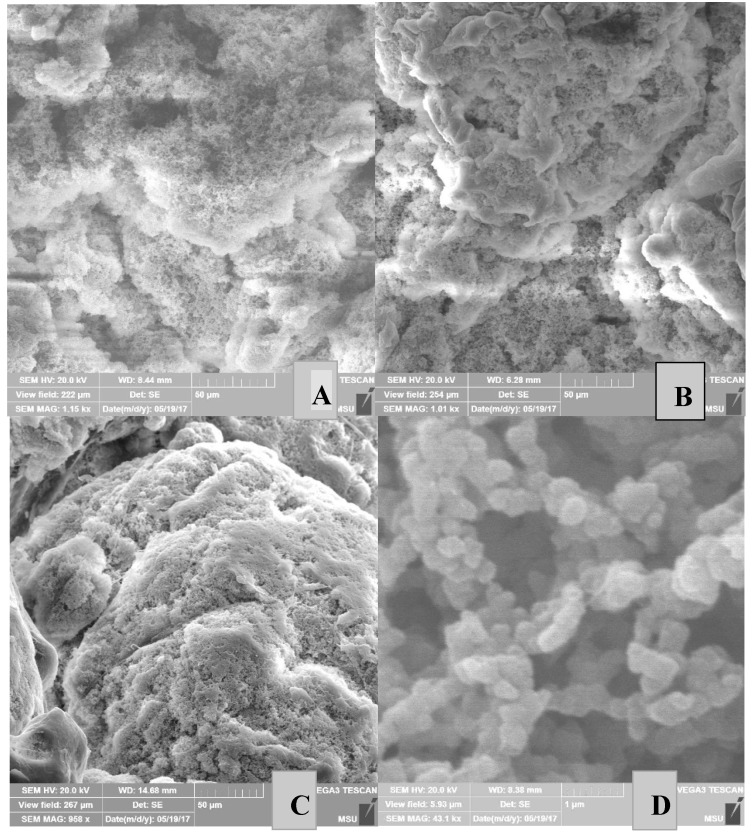

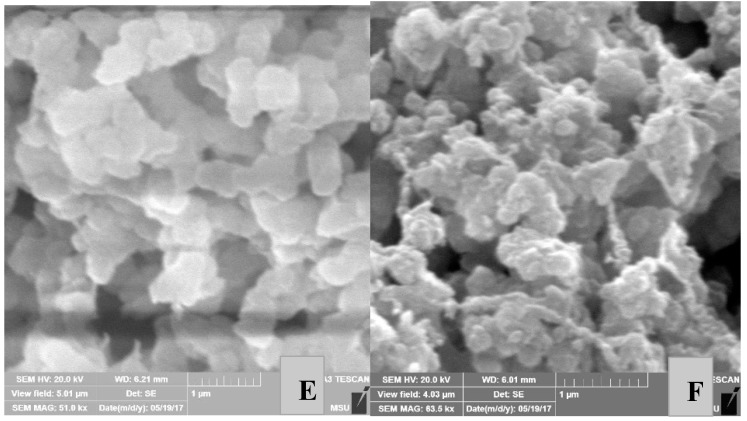
SEM images of the surface morphology of PE powders obtained from catalytic systems **III** in entries 17 (**A**), 19 (**B**), 21 (**C**), 17 (**D**, increase 1 μm), 19 (**E**, increase 1 μm), and 21 (**F**, increase 1 μm).

Figure 5 shows the surface morphology of nascent PE powders obtained from the catalytic system **I** at different polymerization times. As is evident from Figure 5, the PE powder has an irregular surface, which makes it favorable for solvent-free processing. With an increase in the polymerization time, the porous structure of the particles changes, namely, the pore size decreases, and their upper layer is densified. Similar phenomena are observed for nascent PE powders obtained from the catalytic systems **II** and **III** (Figure 6).

The processing of reactor powders of UHMWPE obtained from catalytic systems **I**–**III** into high-modulus oriented films was carried out by preparing monolithic samples under pressure and shear deformation at an elevated temperature below the polymer melting point with subsequent uniaxial stretching by the method described by Ozerin et al. [8].

Table 2 summarizes the set mechanical characteristics of UHMWPE films. It should be noted that these films are characterized by a single-step character of the rupture, which indicates the uniformity of the obtained samples.

**Figure 7:**
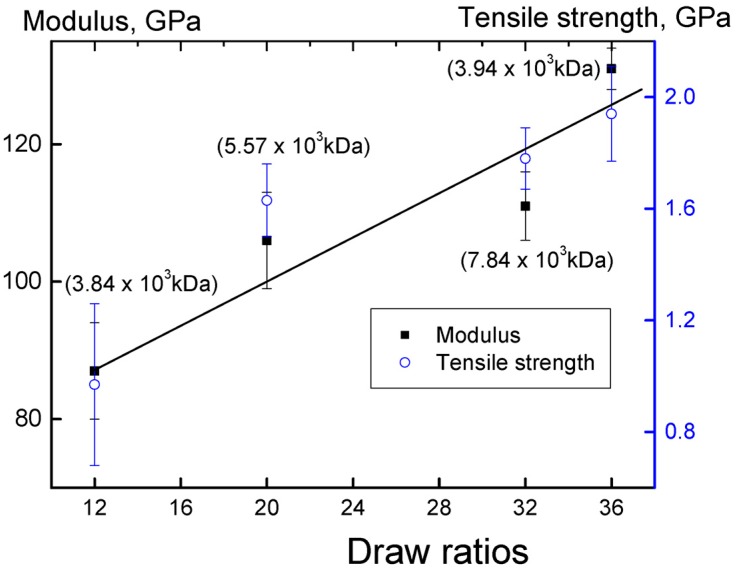
The dependence of the elastic modulus and tensile strength of oriented films on the drawing ratio. Reactor powders obtained from the catalytic system **I** at different polymerization times were used (entries 2–5). Polymer molecular weights are indicated in parentheses.

The best values of the draw ratio as well as the elastic modulus and tensile strength for catalytic system **I** were achieved on the sample obtained after 10 min of polymerization (entry 2). Figure 7 shows the dependence of the elastic modulus and tensile strength of oriented films obtained by solvent-free processing of the reactor powder synthesized in catalytic system **I** on the drawing ratio.

With an increase in the degree of stretching, the strength properties of the oriented films increase linearly, and there is no apparent relationship between the molar masses of the polymer and its mechanical characteristics.

It is known that a solid-phase method can be used to process UHMWPE powders with a porous, “fluffy” particle surface [39]. It is probable that the appearance of fused zones on their surface (Figure 5) with an increase in the polymerization time reduces the quality of the resulting films and prevents the achievement of a high—more than 20-fold—drawing rate.

For correct comparison of the mechanical properties of UHMWPE films obtained from different catalytic systems, we selected samples with the same drawing rate equal to 20 (entries 3, 16–19, 21). As seen from the Table 2 (entries 6 and 16), the samples obtained from catalytic system **II** and from a mixture of TiCl_4_ 2THF + ZrCl_4_ 2THF did not show good mechanical properties, probably due to a long polymerization time. Films from reactor powders synthesized using the TiCl_3_ 2THF are characterized by similar mechanical properties (within the error limits of the corresponding values), which confirms the high quality of the UHMWPE reactor powder of this type. They are inferior in elastic modulus to sample 3, obtained from catalytic system **I**, but superior to the samples obtained from system **II** and the mixed system {TiCl_4_ 2THF + ZrCl_4_ 2THF}.

Uniaxially drawn tapes from UHMWPE powders obtained from a system with Ti(III) significantly outperform the analogues obtained with the participation of Ti(IV) in terms of mechanical properties. 

As shown by the experiments, polymers obtained from catalytic system **I** and from catalytic system **III** with the participation of TiCl_3_·2THF possess the highest drawing ratios. For the samples obtained in entries 2 and 21, the maximum values of the elastic modulus and tensile strength are achieved at the optimum value of elongation at break (Table 2, Figure 7).

## 4. Conclusions

The results indicate the expediency of individual titanium chloride tetrahydrofuran complex application for the formation of THF-containing catalyst systems. Aside from the obvious technological advantages, this approach allows a significant increase in the catalytic activity of systems without any reduction in the mechanical properties of UHMWPE. The introduction of an additional amount of THF into system **II** led to a sharp decrease in its activity.

The catalytic systems containing Ti(III) are, in most cases, inferior in productivity to systems with Ti(IV). This conclusion is consistent with the results reported by Jones et al. [40,41]. A comparison of the productivity of various catalysts for UHMWPE synthesis, including classic Ziegler–Natta (ZN), current commercial generation ZN, metallocene single site, and nonmetallocene catalysts, allowed them to reveal the following activity trend: TiCl_3_ < Mg/Si–TiCl_3_ < metallocenes ≈ nonmetallocenes.

Oriented film tapes from UHMWPE powders obtained from a Ti(III)-containing system noticeably outperform the physicomechanical properties of analogs obtained from a system with TiCl_4_·2THF. This fact confirms the correctness of the assumption that the Ti(IV) atoms are reduced to Ti(III) in systems **I** and **II**, and the fact of increased activity in system **III** in the presence of Et_3_Al indicates that its role is not limited to the reduction reaction alone and that it is apparently necessary for more effective alkylation of the transition metal.

The obtained data on the mechanical characteristics of UHMWPE oriented film tapes with an equal degree of drawing allow us to conclude that the nature of the catalytic system used in the synthesis of the reactor powder affects the strength properties of the filaments. In this case, both the activity of the catalytic system and the *M_W_* of the reactor powder are not among the main factors determining the mechanical properties of the film tapes. Perhaps the nature of the catalytic system, influencing the morphology of the reactor powder, indirectly predetermines the topology of the fibrillar structure, which is formed during the solid-phase forming of films and their subsequent uniaxial drawing. Its study, depending on the nature of the catalyst, requires additional research and is beyond the scope of this paper.

## Figures and Tables

**Table 1:** Ethylene polymerization results using catalytic systems **I**–**III** ^a^.

Entry	Catal. system	(Ti), mol	Activators, (Et_2_AlCl):(Et_3_Al):(Bu_2_Mg)	*t*, min	*m_polym_*, g	A ^b^	*T_m_* ^c^	Degree of crystal., ^c^ %	Bulk density, g/cm^3^	*M_W_* 10^6^ Da
1	**I**	2 × 10^−5^	300:40:30	5	2.10	1260	144	77	0.06	1.8
2	**I**	2 × 10^−5^	300:40:30	10	4.20	1260	144	76	0.07	3.8
3	**I**	2 × 10^−5^	300:40:30	15	5.00	1000	146	76	0.06	5.6
4	**I**	2 × 10^−5^	300:40:30	30	7.20	720	144	71	0.07	7.8
5	**I**	2 × 10^−5^	300:40:30	60	12.00	600	144	74	0.05	3.9
6	**II**	1 × 10^−5^	300:40:30	30	4.10	820	146	77	0.05	3.3
7	**II**	1 × 10^−5^	300:40:30	30	9.20	1840	141	63	0.07	7.2
8	**II** ^f^	1 × 10^−5^	300:40:30	30	1.50	300	148	78	0.07	6.2
9	**II**	1 × 10^−5^	150:40:30	30	1.41	282	142	61	0.09	2.9
10	**II**	1 × 10^−5^	300:0:30	30	8.34	1668	143	66	0.07	3.9
11	**II**	1 × 10^−5^	150:0:50	30	6.60	1320	142	69	0.06	5.7
12	**II** ^g^	1 × 10^−5^	150:0:50	30	4.74	948	144	69	0.09	n.d
13	**II** ^d^	1 × 10^−5^	300:0:30	30	1.15	282	137	70	0.31	1.1
14	**II** ^e^	1 × 10^−5^	300:0:30	30	0.34	68	140	45	0.45	n.d
15	Ti–Zr	1 × 10^−5^ + 1 × 10^−5^	300:40:30	30	3.00	600	142	58	0.06	n.d
16	Ti–Zr	1 × 10^−5^ + 1 × 10^−5^	600:80:60	30	5.96	1192	142		0.05	3.7
17	**III**	1 × 10^−5^	300:40:30	30	6.97	1420	144	77	0.06	2.2
18	**III** ^g^	1 × 10^−5^	150:0:50	30	4.83	966	142	67	0.06	6.3
19	**III**	1 × 10^−5^	300:0:30	30	3.64	728	144	69	0.07	6.3
20	**III**	1 × 10^−5^	300:0:100	30	2.70	540	149	78	0.09	3.8
21	**III**	1 × 10^−5^	150:0:50	30	4.78	956	143	67	0.06	2.5
22	**I** ^d^	2 × 10^−5^	300:0:30	30	1.95	286	139	58	0.26	0.8
23	**I** ^e^	2 × 10^−5^	300:0:30	30	0.40	68	140	45	0.38	0.7

^a^ Polymerization carried out in 50 mL of petroleum ester (entries 1–6) or toluene (entries 7–23) at a constant 0.7 atm ethylene pressure, temperature 30 °C; ^b^ A-activity is expressed as kg PE/mol Ti·h·atm; ^c^ Melting points and degree of crystallinity were determined by DSC; ^d^ methylaluminoxane (MAO) instead of Et_2_AlCl; ^e^ iBu_3_Al instead of Et_2_AlCl; ^f^ 300:0:30 with additional 80 equiv. THF; ^g^ Et_3_Al_2_Cl_3_ instead of Et_2_AlCl.

**Table 2:** Mechanical properties of monolithic UHMWPE tapes.

Entry ^a^	Catalytic system	*M_W_* (10^6^)	Elongation, %	Drawing ratio	Breaking strength, GPa	Elastic modulus, GPa
1	**I**	1.83	2.4		1.2	60
2	**I**	3.84	2.0	36	2.1	125
3	**I**	5.57	2.1	20	1.6	106
4	**I**	7.84	2.2	32	1.9	100
5	**I**	3.94	2.1	12	1.2	60
6	**II**	3.33	3.0	12	1.2	45
16	Ti–Zr	3.70	3.2	20	1.0	44
17	**III**	2.15	2.7	20	1.8	78
18	**III**	6.28	2.7	20	1.8	78
19	**III**	6.28	2.3	20	1.6	81
20	**III**	3.79	2.9	24	2.0	70
21	**III**	2.47	2.5	20	1.8	88

^a^ Numbering corresponds to Table 1.
